# Effect of integrated yoga practices on immune responses in examination stress – A preliminary study

**DOI:** 10.4103/0973-6131.78178

**Published:** 2011

**Authors:** Aravind Gopal, Sunita Mondal, Asha Gandhi, Sarika Arora, Jayashree Bhattacharjee

**Affiliations:** Department of Physiology, Lady Hardinge Medical College, New Delhi, India; 1 Department of Biochemistry, Lady Hardinge Medical College, New Delhi, India

**Keywords:** Cell-mediated immune responses, IL-4, IFN-γ, state anxiety score; stress, yoga

## Abstract

**Background:**

Stress is often associated with an increased occurrence of autonomic, cardiovascular, and immune system pathology. This study was done to evaluate the impact of stress on psychological, physiological parameters, and immune system during medical term -academic examination and the effect of yoga practices on the same.

**Materials and Methods:**

The study was carried out on sixty first-year MBBS students randomly assigned to yoga group and control group (30 each). The yoga group underwent integrated yoga practices for 35 minutes daily in the presence of trained yoga teacher for 12 weeks. Control group did not undergo any kind of yoga practice or stress management. Physiological parameters like heart rate, respiratory rate, and blood pressure were measured. Global Assessment of Recent Stress Scale and Spielbergers State Anxiety score were assessed at baseline and during the examination. Serum cortisol levels, IL-4, and IFN-γ levels were determined by enzyme-linked immunosorbent assay technique.

**Result:**

In the yoga group, no significant difference was observed in physiological parameters during the examination stress, whereas in the control group, a significant increase was observed. Likewise, the indicators of psychological stress showed highly significant difference in control group compared with significant difference in yoga group. During the examination, the increase in serum cortical and decrease in serum IFN-γ in yoga group was less significant (*P*<0.01) than in the control group (*P*<0.001). Both the groups demonstrated an increase in serum IL-4 levels, the changes being insignificant for the duration of the study.

**Conclusion:**

Yoga resists the autonomic changes and impairment of cellular immunity seen in examination stress.

## INTRODUCTION

Stress may be defined as psychophysiological process usually experienced as a negative emotional state. It is a common condition, a response to a physical threat or psychological distress that generates a host of chemical and hormonal reactions in the body. The health effects of stress involve mainly autonomic, cardiovascular, and immune systems.[1]

Studies have shown that stress is associated with increased latent viral reactivation,[2] upper respiratory tract infection,[3] and wound-healing time,[4,5] indicating that stress causes significant immune response dysfunction. George F. Solomon first demonstrated the influence of stress on immune response in animals and human beings in 1964.[6] Academic stress, the stressful condition of students taking examination, can be considered as a good model of naturalistic stress in human beings as compared with laboratory-induced stress situations.[7] According to Herbert and Cohen classification, it is an objective, discrete, short-term, and nonsocial stress.[8] Danner *et al*.[9] and Shukla *et al.*[10] in their studies on examination stress in medical students have demonstrated significant increase in the pulse rate and blood pressure during examinations as compared with baseline nonstressful states. Examination stress has consistently shown to cause changes in catecholamine levels, whereas the changes in cortisol and Adrenocorticotropic Hormone (ACTH) were somewhat contradictory, with some studies showing an increase[11,12] and others showing no relation.[13,14]

In response to stress, activation of Hypothalamo-Pituitary-adrenal Axis (HPA) results in secretion of Corticotrophin-Releasing Factor (CRF) from hypothalamus. CRF stimulates the secretion of ACTH from pituitary, which further activates the adrenal glands to produce glucocorticoids, which are powerful immune regulators.[15] The effects of glucocorticoids on cellular and humoral immune responses is quite complex. Although the overall effect of glucocorticoids on immune/inflammatory responses at the cellular level is immunosuppressive, this effect may result from suppression of many stimulatory components of immune cascade and stimulation of some immunosuppressive or anti-inflammatory elements. The relatively greater sensitivity of components of cellular immunity to glucocorticoid suppression tends to shift immune response from a cellular to humoral pattern during stress.[16]

There is preliminary evidence that relaxation therapy may influence endocrine function and counter balance stress-induced endocrine changes.[17] Stress management is required for medical students to decrease depression and anxiety, and to improve sensitivity toward themselves, peers, and parents.[18,19] Several stress management programs, e.g., meditation, yoga, hypnosis, imagery, muscle relaxation, have been introduced in the past. Yoga is an ancient science established in India, which gives the practitioner not only a healthy body but also a sound mind. Yoga has been shown to have effects on most physiological systems of the body. Among the various biochemical effects of yoga, a decrease in the cortisol levels[20] and control of glucose levels in diabetic patients[21] have been reported. Earlier studies in yoga in medical students have pointed toward the role of yoga in stress management, but none of the studies have reported the effect of Yoga on immune changes that occur with examination stress. This study addresses the effect of examination stress on immune function and how yoga influences those immune changes.

## MATERIALS AND METHODS

The study was carried out on sixty first-year MBBS student volunteers staying in hostel/campus, who did not suffer from any acute or chronic physical illness. Our medical college offers MBBS courses only for girl candidates; hence, all of the students selected were young girls of age 17 to 20 years. These students were randomly assigned to yoga group and control group (30 each). All of the students were assessed twice during the study—the first, at the time of enrolment (baseline levels when no examination stress was there) and the second, 3 months later during their exams (exam stress). The 30 students included in yoga group underwent integrated yoga practices for 35 minutes daily in the presence of a trained yoga teacher for 12 weeks. The control group followed their normal daily routine and did not practice any of the yogic techniques practiced by yoga group.

The following yogic techniques were daily practiced by the subjects of yoga group:

- Yogic prayer 2 minutes
- *Sukshma Vyayam* (micro exercises) 6 minutes
- *Sthula Vyayama* (macro exercises) 4 minutes
- Asanas (postures) 12 minutes
- Pranayama 4 minutes
- Dhyana (meditation) 5 minutes

### Physiological parameters

Physiological parameters like Heart Rate and Respiratory rate were assessed by using Biopac physiology lab version 3.0 after proper calibration. Blood pressure was measured by sphygmomanometer.

Rate pressure product was calculated by multiplying the systolic blood pressure with the heart rate and then dividing it by hundred. It is a sensitive index of myocardial oxygen consumption.

### Psychological assessment

#### Global assessment of recent stress scale

Global Assessment of Recent Stress Scale (GARS) is a reliable and valid method to estimate present level of perceived short-term stress. It allows evaluation of the amount of stress thought as a feeling of pressure one has been under in a well-defined time (e.g., the previous week). It consists of seven areas (scores 0-9). The students were asked to think of a time when they were under stress and describe it and to compare it with how they felt when they were free from worries, pressures, and stress. The subjects were then asked to think of the past week and describe the stress as “pressure” on a scale of 0-9 (none to severe). The seven areas of life included were work pressure, interpersonal relationship, changes in relationships, sickness or injury, financial issues, unusual happenings, change or lack of change from routine. Total scores were made by adding up individual scores that the students had marked in the questionnaire.[22]

#### State trait anxiety inventory for adults

The state trait anxiety inventory for adults (STAI-A) is the definitive instrument for measuring anxiety in adults. It clearly differentiates between the temporary condition of “state anxiety” and the more general and long-standing quality of “trait anxiety.” The STAI has forty questions with a range of four possible responses to each. State anxiety may fluctuate over time and can vary in intensity. In contrast, trait anxiety denotes “relatively stable individual differences in anxiety proneness” and refers to a general tendency to respond with anxiety to perceived threats in the environment. At the end of the study period, the questionnaires were scored by adding the weighted (1 to 4) scores of each item, using the directions and scoring key provided in the Manual for State-Trait Inventory (Form Y).[23] The scores could vary from a minimum of 20 to a maximum of 80. The scoring of the STAI scale was done by an investigator who had no knowledge of the study protocol, and who was also blinded to other subject-related study data. State Anxiety scores of subjects undergoing Yoga were compared with controls, and baseline scores were compared with the stress period (examination).

#### Collection of blood sample for biochemical parameters

All of the subjects were asked to report at 8 am. Taking all aseptic precautions, 5 ml venous blood sample was drawn from the antecubital vein of each subject on both the occasions of blood sampling (at enrolment and during stress). The serum separated after centrifugation was divided into aliquots and batch analyzed by Enzyme-Linked Immunosorbent Assay (ELISA) after every collection.

### Biochemical parameters

#### Serum cortisol levels

Serum cortisol was estimated by solid-phase competitive ELISA using a commercial kit (EIAgen cortisol, Adaltis Italia, Italy).

#### Immunological parameters

Serum Interleukin (IL-4) was estimated by solid-phase sandwich ELISA using commercial kit (Diaclone Research, France).

Serum Interferon (IFN-γ) was estimated by solid-phase sandwich ELISA using commercial kit (Diaclone Research, France).

### Statistical analysis

The statistical analysis was done using SPSS for windows 10.0 software (SPSS Inc., Chicago, IL, USA). Data are expressed in mean±standard error of mean. Intra-group and inter-group comparison of physiological parameters and biochemical parameter (serum cortisol) was done using paired *t*-test. For nonparametric data, such as levels of IL-4 and IFN-γ, intragroup comparison was done by Wilcoxon Signed Rank test, and for inter-group comparison, Mann Whitney *U* test was used.

## RESULTS

The students selected in the study and control group were matched for age, height, and weight. Baseline values of heart rate, blood pressure, respiratory rate, rate pressure product, serum cortisol, IL-4, and IFN-γ did not show significant difference between the Yoga group and the control group.

## Changes in physiological parameters with examination stress

A highly significant increase was seen in heart rate and mean rate pressure product and significant increase was seen in systolic blood pressure and mean rate pressure product in control group. However, no significant difference from baseline values was seen in the Yoga Group. There was no significant difference in diastolic blood pressure in any of the group during examination stress [Table 1]

**Table 1:** Changes in physiological parameters with examination stress

	Baseline (±SEM)	Examination stress (±SEM)	*P*-value (baseline vs exam stress)
Heart rate (beats/Min)			
Control group (n=30)	80.93±1.23	85.6±1.21	*P*<0.001
Yoga group (n=30)	82.67±1.63	80.80±1.16*	*P*=0.105
Systolic blood pressure (mm Hg)			
Control group (n=30)	119.33±1.42	122.93±0.90	*P*=0.012
Yoga group (n=30)	119.73±1.41	120.20±1.06	*P*=0.774
Diastolic blood pressure (mm Hg)			
Control group (n=30)	77.2±1.05	78.2±0.76	*P*=0.326
Yoga group (n=30)	76.47±1.17	75.47±0.92	*P*=0.477
Mean rate pressure product			
Control group (n=30)	96.45±1.64	105.15±1.49	*P*<0.001
Yoga group (n=30)	99.09±2.46	97.22±1.82*	*P*=0.689
Respiratory rate (cycles/Min)			
Control group (n=30)	16.03±0.52	16.77±0.44	*P*=0.019
Yoga group (n=30)	17.33±0.60	16.67±0.43	*P*=0.132

Significant difference on intergroup comparison is designated by

* if *P*<0.05

** if *P*<0.01

## Changes in psychological parameters with examination stress

GARS scores which signified the level of stress perceived by the student over the preceding week was found to increase in the control group, whereas a highly significant decrease was observed in the Yoga group [Table 2]. The GARS score was comparable in both the groups at the beginning of the study. However, during the examination, GARS score was significantly lower in Yoga group as compared with the control group.

When the Spielbergers State Anxiety Scores were compared at the baseline, no significant difference was observed between the Yoga and the control group. The STAI scores were found to increase in both the study and the control group, but the increase in the control group was more in magnitude and very highly significant as compared with the Yoga group [Table 2]. During examination, STAI scores were significantly higher in control group as compared with Yoga group (*P*<0.05).

**Table 2:** Changes in psychological parameters with examination stress

Parameter	Baseline (±SEM)	Examination stress (±SEM)	*P*-value (baseline vs exam stress)
GARS score			
Control group (n=30)	23.90±7.64	28.61±8.72	*P*<0.001
Yoga group (n=30)	27.73±8.19	23.20±8.16*	*P*<0.01
STAI scores			
Control group (n=30)	43.07±9.01	55.93±9.35	*P*<0.001
Yoga group (n=30)	45.37±9.01	48.87±12.81*	*P*<0.05

Significant difference on intergroup comparison is designated by

* if *P*<0.05

** if *P*<0.01

## Changes in serum cortisol with examination stress

Both the study group and the control group showed a significant increase in serum cortisol levels during examination stress when compared with their baseline values. However, the observed increase in control group was higher (187.16%) and statistically more significant (*P*<0.001) than in the Yoga group (93.1% increase, P<0.01) [Table 3]. On intergroup comparison, there was an insignificant difference in cortisol levels at the beginning of the study; however, during examination, the mean cortisol levels were significantly higher in control group as compared with Yoga group [Table 3].

**Table 3:** Changes in biochemical and immunological parameters with examination stress

Parameter	Baseline (±SEM)	Examination stress (±SEM)	*P*-value (baseline vs exam stress)
Serum cortisol levels (ng/ml)			
Control group (n=30)	121±11.5	281.77±17.4	P<0.001
Yoga group (n=30)	119.9±6.2	231.63±14.1*	P=0.008
Serum IL-4 (mg/ml)			
Control group (n=30)	1.04±0.319	1.05±0.25	P=0.99
Yoga group (n=30)	0.91±0.18	0.98±0.19	P=0.638
Serum IFN-γ (pg/ml)			
Control group (n=30)	4.29±0.902	1.87±0.241	P=0.012
Yoga group (n=30)	5.18±1.39	4.58±1.62*	P=0.794
Serum IFN-γ/IL-4 ratio			
Control group (n=30)	4.15±3.12	1.78±0.91	P=0.008
Yoga group (n=30)	5.69±4.16	4.67±3.21*	P=0.321

Significant difference on intergroup comparison is designated by

* if P<0.05

** if P<0.01

## Changes in serum IL-4 with examination stress

Both the study group and the control group showed a slight increase in serum IL-4 levels during examination stress when compared with their baseline values. However, the observed difference was insignificant for the duration of the study, indicating an increase in humoral immunity during examination stress [Table 3].

## Changes in serum IFN-γ with examination stress

Serum IFN-γ levels decreased in both the Yoga group and the control group; however, the decrease was found to be significant in the control group but insignificant in the Yoga group [Table 3]. At baseline, the IFN-γ levels were comparable in both the groups but were significantly higher in Yoga group as compared with control group during examinations.

## Changes in serum IFN-γ/IL-4 ratio

Serum IFN-γ/IL-4 ratio was comparable in both the groups at the beginning of the study. However, this ratio decreased significantly in control group, whereas the decrease in yoga group was insignificant. During examination, serum IFN-γ/IL-4 ratio was significantly higher in yoga group as compared with control group [Table 3].

## DISCUSSION

The present study was conceived with the intention of studying the effect of integrated yoga practices on immune responses in examination stress. It also addressed the effects of examination stress on autonomic and endocrine variables and the effect of yoga practices on the same.

In our study, it was observed that the control group students (not practicing yoga) had a 5.98% increase in heart rate, a significant increase in systolic blood pressure (*P*<0.05), increase in diastolic blood pressure by 1.67% (though not significant), an increase in rate pressure product (*P*<0.001), increase in respiratory rate (*P*<0.05) during examination stress. On the contrary, students practicing Yoga demonstrated a decrease in heart rate by 1.74%, with no significant increase in systolic and diastolic blood pressures, a decrease in rate pressure product though not significant, and a decrease in respiratory rate (not statistically significant) during examination stress as compared with baseline.

Our observation in the control group is in concordance with earlier studies, wherein Lovallo *et al*. reported an increase in heart rate, a nonsignificant increase in systolic blood pressure, and an increase in rate pressure product.[24] Other researchers also reported a similar trend of increasing pulse rate, blood pressure, and galvanic skin resistance during examination stress.[25,26] Our study on effects of yoga are in concordance with the earlier studies by Hoenig where he showed a decrease in heart rate and blood pressure and proposed the mechanism to be that of Valsalva Manoeuver.[27] Several workers have proposed that the decrease in heart rate and blood pressure with Yoga is most likely due to inhibition of posterior or sympathetic area of hypothalamus leaving the parasympathetic area alone, thus decreasing the sympathetic activity without affecting parasympathetic activity.[28–30]

The GARS score was found to decrease in yoga practicing students (15.20%), whereas in control group, a 21.61% increase was observed. The State anxiety levels which indicate the anxiety a person feels at a particular point of time was seen to increase both in Yoga group (11.38%) and control group (31.56%). Thus, the observed increase in state anxiety levels was higher in the control group. Examination stress is expected to increase the anxiety and almost all studies done on examination stress are in concordance with this.[31–33] The effect of Yoga on anxiety has been thoroughly studied and reported by various investigators, wherein significant decrease has been demonstrated in STAI scores with yoga.[26,34] The decrease in anxiety levels seen in GARS signifies that yoga has a calming effect on the mind and body while the student is passing through a stressful period. But the mild increase in anxiety scores in STAI at the start of examination when compared with controls signifies that when the challenge is in front of them, they just react like the controls but to a lesser degree. The subjective feeling of anxiety is thus tempered down by Yoga. Probably, an optimum level of anxiety is required to arouse a person to face a challenge and to perform well. Yoga keeps the anxiety levels in check such that it rises only to level where it is beneficial and not harmful.

All of the students in the study as well as the control group demonstrated a significant increase in serum cortisol levels during examination as compared with their baseline levels; however, the increase in control group were higher and more significant than those observed in the study group. The increase in cortisol levels was expected since cortisol is one of the main hormones involved in stress response. The emotions directly stimulate the limbic system which has profound effects on the hypothalamus to release ACTH, which stimulates the adrenal cortex to secrete cortisol. Similar increase in cortisol levels immediately before and during the examinations has also been reported by various researchers,[24,35,36] whereas other researchers like Glaser *et al*.[14] and Semple *et al*.[13] did not find any change in cortisol levels during examination. The findings of the present study clearly indicate that yoga practice attenuates the increase in cortisol levels during examination stress. This attenuation can be attributed to the relaxing effect of yoga on mind, which decreases the stimulus to the hypothalamus and hence anterior pituitary, resulting in lower cortisol levels. Schmidt *et al*.[37] and Kamei *et al*.[20] have also demonstrated a fall in cortisol levels with yoga practice.

The present study has showed that the mean plasma IL-4 levels rise and serum IFN-γ levels decrease with examination stress. Of this, the decrease in serum IFN-γ in control group was statistically significant (*P*<0.01), whereas decrease in yoga group was not significant. The decrease in serum IFN-γ indicates a decline in cellular immunity with examination stress. However, yoga seems to have some buffering effect on the impairment of cellular immunity as observed from IFN-γ levels of the yoga group during examination stress. From the observed changes in the cytokine levels (IL-4 and IFN-γ) during the examination stress, it appears that Th1 cell activity decreases significantly while Th2 activity remains more or less the same during examination stress. Paik *et al*. also showed that IL-4 levels increased and IFN-γ levels decreased during examination stress and postulated that immune system biases to Th2 cell activity during examination stress.[38] Halvorsen and Vassend[39] studied IL-2 receptor activity as an index of Th1 activity and reported a decrease in activity during examination stress. Dobbin *et al*.[40] also showed that lymphocyte responsiveness decreased, IFN-γ decreased, but IL-1g increased after examination. The adrenal steroids have been postulated to play an important role in trafficking of immune cells[41] and also increase humoral immunity and decrease cellular immunity.[42]

The buffering effect of yoga on stress-induced decrease in cellular immunity may be due to its ameliorating action on HPA, creating an optimized secretion of cortisol. Yoga also restores the autonomic reflex regulatory mechanisms, creating a balance between sympathetic and parasympathetic limbs in the presence of a stressor which probably prevents the impairment of cell-mediated immunity as reflected in IFN-γ levels.

This study is, however, a preliminary study focusing on a cytokine each from humoral and cell-mediated immune system. Further large-scale studies focusing on the effect of Yoga on complete cytokine profile or analyzing the effect on various immune effector cells (dendritic cells, natural killer cells, cytotoxic-T cells) would provide a more definitive view of the role of Yoga and other relaxation therapies on the immune system.

## CONCLUSION

Thus, authors conclude that yoga has a significant effect in ameliorating the autonomic, endocrine, and psychological changes brought about by the examination stress. Yoga most probably acts through the cerebro-cortico-limbic pathways on the hypothalamus and the anterior pituitary systems. It thus influences the HPA in such a way that the activation of this system is optimized and a balance is created between the sympathetic and parasympathetic limbs of the autonomic nervous system when the subject is faced with a threat (which in present case is the examination stress). Hence, the subjects practicing yoga do not show as much increase in autonomic variables as seen in the control group. In addition, the circulating levels of cytokines suggest that yoga also has a beneficial effect on the immune system of the individual.
